# Welfare Assessment of 30 Dairy Goat Farms in the Midwestern United States

**DOI:** 10.3389/fvets.2021.646715

**Published:** 2021-04-30

**Authors:** Melissa N. Hempstead, Taylor M. Lindquist, Jan K. Shearer, Leslie C. Shearer, Vanessa M. Cave, Paul J. Plummer

**Affiliations:** ^1^Veterinary Diagnostic and Production Animal Medicine, College of Veterinary Medicine, Iowa State University, Ames, IA, United States; ^2^Knowledge and Analytics, AgResearch Ltd., Ruakura Research Centre, Hamilton, New Zealand

**Keywords:** animal welfare, animal husbandry, welfare assessment, well-being, goat, caprine, dairy

## Abstract

Dairy goat animal welfare assessment protocols have been developed and conducted in Europe and the United Kingdom for dairy goats; however, there are no published reports of large-scale welfare assessment for dairy goats on farms in the Midwestern United States (US). Therefore, the objective of this study was to perform welfare assessment of lactating dairy goats and identify the most prevalent welfare issues on 30 farms across the Midwestern US. Thirty dairy goat farms (self-selected) were enrolled in the study if they shipped milk for human consumption (regardless of herd size). The number of lactating does on each farm ranged from 34 to 6,500 goats, with a median number of 158 lactating does (mean ± SD: 602 ± 1,708 lactating does). The protocol used was developed from available literature on goat welfare assessment but modified for use in the Midwestern US. Observations were made without handling the animals and included 22 animal-based indicators evaluated at the group- and individual-level. The observations were conducted during ~3–5 h during a milking session (either morning or afternoon) and time in the home pen. Principal components analysis (PCA) was carried out on the welfare assessment data from each farm. The first two dimensions of the PCA explained 34.8% of the variation. The PCA biplot indicated correlations between indicators. The most prevalent conditions observed across the 30 farms included any knee calluses (80.9%), any claw overgrowth (51.4%), poor hygiene (14.9%), skin lesions (8.9%), poor hair coat condition (8.3%) and any ear pathology (8.0%). These results are the first to provide the Midwestern US dairy goat industry with information to improve commercial dairy goat welfare.

## Introduction

Defining animal welfare is difficult because there are multiple interpretations (1). An early interpretation of animal welfare was formulated by the Farm Animal Welfare Council, named the “Five Freedoms,” and outlined the basis of acceptable levels of welfare (i.e., freedom from hunger or thirst, discomfort, pain, injury or disease, fear and distress and the freedom to express normal behaviors (2). Since then, other viewpoints have been developed such as the “three overlapping dimensions” of welfare where an animal's quality of life relates to basic health and functioning, affective states, and natural living (3), or the “Five Domains” model, whereby an animal experiences good welfare if its nutritional, environmental, health, behavioral, and mental (i.e., affective state) needs are met (4). However, regardless of how animal welfare is defined, the development of an on-farm monitoring system or welfare assessment protocol, which encompasses multiple indicators of welfare can be developed and utilized for small ruminants (5).

Early research on development of protocols to assess welfare at the farm-level for dairy goats evaluated multiple animal-based indicators of welfare and highlighted the major welfare issues across 24 farms in the UK (6) and 30 farms in Norway (7). Since then, the European Animal Welfare Indicators Project (AWIN) developed a science-based, step-wise welfare assessment protocol for species (including goats, sheep, horses, donkeys, and turkeys) that had until then, been largely excluded from welfare assessment projects such as Welfare Quality® (8). Welfare Quality®, a large-scale science-based European program designed to assess the welfare of cattle, swine, and poultry used a framework consisting of 4 key principles (i.e., good feeding, housing and health, and appropriate behavior), with 12 criteria (e.g., absence of prolonged hunger, comfort around resting, expression of social behavior) (9). AWIN was based on the same such principals and criteria as Welfare Quality® as they are considered necessary to cover all aspects of animal welfare (8). Some examples of animal-based indicators of welfare used by AWIN include hair coat and body condition, fecal soiling, udder asymmetry, overgrown claws, and lameness (10). Development and testing of the AWIN protocol for dairy goats has since demonstrated valid, reliable, and feasible animal-based indicators of welfare in a European setting (11–15). However, to the authors' knowledge, no such on-farm welfare assessment protocols have been designed for, or undertaken on dairy goats in the Midwestern US.

In the US, there are welfare assessments of commercial swine [see review by (16)], poultry [see review by (17, 18)], dairy cattle (19) and turkey (20) farms. However, welfare assessment data for dairy goats in the US is scarce. In 2020, there were ~440,000 dairy goats in the US, and of those, 135,000 (~31%) were populated in the Midwestern region comprising Minnesota, Iowa, Wisconsin, and Illinois (21). Dairy goat welfare assessment data can help inform producers on areas of deficiency and consequent improvement, promotion of good welfare policies, and can add to the growing body of science-based research on welfare assessment of dairy goats worldwide.

The objective of this study is to perform welfare assessment of dairy goats and identify the most prevalent welfare issues on 30 farms across the Midwestern United States (US).

## Materials and Methods

This study was approved by the Institutional Animal Care and Use Committee at Iowa State University prior to data collection (Protocol number: IACUC-18-341).

### Farm Recruitment

Advertising material was distributed to farms by a milk company operating in the Midwestern region on our behalf. Additionally, farms were visited by study personnel (with a feed representative) and advertising material was distributed directly to farm owners. Participation was incentivized by receipt of compensation associated with participation on the study. Once 30 dairy goat farm owners had voluntarily completed an online application form (Smartsheet Inc., Bellevue, WA), their farms were enrolled in the study if they shipped milk for human consumption (regardless of herd size) and were situated within the Midwestern states: Minnesota, Iowa, Wisconsin, and Illinois. Farm owners were asked to complete a survey independently of on-farm assessment, which focused on farm owner attitude to goat behavior and welfare, husbandry practices, goat-specific information and other details of the farm (Hempstead et al., unpublished data).

### Protocol Development

The protocol was developed from the available literature on goat welfare assessment (5, 10, 22) including assessment protocols that had been used previously (6–8, 12, 14). The protocol was designed for use on adult lactating does and comprised 22 animal-based indicators of welfare at the individual- (9 indicators; Table 1) and group-level (13 indicators; Table 2) that were decided for inclusion by a small committee of veterinary practitioners and an animal scientist.

**Table 1:** Descriptions of the *individual-level* welfare indicators and the order of which they were assessed for the dairy goat welfare assessment protocol.

**Order**	**Welfare indicator**	**Description**
1	Ear pathology	
	Ear tear Missing ear tag Infected ear tag Frostbitten ears	Complete or partial tear of the pinna. A hole in the pinna from a missing ear tag. Ear tag with evidence of infection (e.g., swelling, pus). Any amount of pinna is missing (appears as a straight cut).
2	Ocular discharge	Moist (or dry) fluid from the eye(s) that is clear or colored fluid, thick, or runny.
3	Nasal discharge	Moist (or dry) fluid from the nostril(s) that is clear or colored fluid, thick, or runny.
4	Skin lesion	Any broken skin, abscess or ulceration (fresh or in the process of healing, i.e., crust). Regions that were observed for skin lesions included the head or neck, and the rump or thigh. Fully re-epithelialized tissue was excluded.
5	Knee callusing	
	Mild	Thickened skin (with hair loss) covered *part* of the knee. The score of the worst knee was recorded. Knees were not scored if calluses were not clearly visible (i.e., too dirty).
	Severe	Thickened skin covered the *entire* knee (with hair loss) and may have had broken skin. The score of the worst knee was recorded. Knees were not scored if calluses were not clearly visible (i.e., too dirty).
6	Poor hygiene	The presence of any fecal material (or dirt) on the hind quarters (i.e., rump, thigh, rear legs, udder) that can be dry or moist. Goats that kidded recently (i.e., visible afterbirth or blood) were not scored.
7	Fecal soiling	Presence of feces around the anus or sides of the tail. Goats that kidded recently (i.e., visible afterbirth or blood) were not scored.
8	Udder asymmetry	One side of the udder was >25% longer than the other side (from the udder attachment to udder floor; excluding teat).
9	Overgrown claw	Only the rear claws were assessed.
	Mild	Overgrowth beyond the triangular shape of the claw, but no change in hoof conformation. The score of the worst claw was recorded.
	Severe	Extreme claw overgrowth with loss of the triangular shape and conformational changes of the hoof, which may include weight bearing on the heel. The score of the worst claw was recorded.

*Both sides of the animal were assessed for all indicators*.

**Table 2:** Descriptions of the *group-level* welfare indicators and the order of which they were assessed for the dairy goat welfare assessment protocol.

**Order**	**Welfare indicator**	**Description**
1	Queuing at feed rack(s)	Goat standing behind another goat at the feed rack(s) within 1 m with head oriented toward the feed rack(s) during feeding time. The number of goats queuing was counted over 16 min (scan sample every 2 min).
2	Queuing at drinking place(s)	Goat standing behind another goat at the drinking place(s) within 1 m with head oriented toward the drinking place. The number of goats queuing was counted over 16 min (scan sample every 2 min) during feeding time.
3	Horn growth	
	Horn	Horn(s) with normal growth. Horns with the tip mechanically removed were included.
	Scur	Soft, partially formed horn that is not attached to the underlying frontal bone.
4	Poor hair coat condition	Dull, rough, and shaggy hair coat that may be longer on some parts of the body than others.
5	Thermal stress	
	Cold	Cramped posture (arched back) raised hair along the neck and spine (i.e., horripilation), limited movement and may include shivering. Goats that were involved in agonistic interactions (with associated horripilation) were excluded.
	Heat	Accelerated respiration rate, open mouth panting with or without drool from the lips.
6	Kneeling	Transitions between lying and standing were excluded.
	In pen At feed rack(s)	Knees touching the pen floor at the *lying area* (≥5 s/bout). Knees touching the pen floor at the *feed rack* (≥5 s/bout).
7	Latency to approach test	Time taken for a goat to contact any part of a novel person in the pen (including clipboard). The assessor moved to a predetermined location in the pen, usually with their back to the wall or gate. The test ended after a non-contact time of 5 min.
8	Body condition	
	Overweight	Hip and pin bones were difficult to identify and the line between them was convex.
	Underweight	Hip and pin bones were prominent and the line between them was concave.
9	Lameness	Abnormal gait and curvature of the spine that may have included head nodding (bobbing). Goats were encouraged to walk by the assessor. Those that did not stand or had any obvious injuries were excluded.

Sampling periods included (1) assessments of individuals in the milking parlor during routine milking and (2) group assessments, which were carried out in the home pen. The order of these sampling periods (i.e., at milking or the home pen), depended on whether a morning or afternoon milking session was attended. Within each sampling period, the indicators were assessed in the same order for each farm (Tables 1, 2); for example, if the morning milking session was observed (between 0400 and 0700 h), then the group-level assessment took place following milking. However, if an afternoon milking session was observed (between 1400 and 1800 h), the group-level assessment was carried out prior to milking. The separate sampling periods were chosen in order to facilitate multiple farm visits within 1 day. The time of feed distribution relative to assessment of the home pen was not recorded. Observations were performed without animal handling. Indicators were excluded if they (i) required laboratory analysis, or specific instruments to be used on the animal (e.g., stethoscope, thermometer), (ii) were overly time consuming and could not be carried out on the day of observation (i.e., requiring post-observation video analysis), (iii) were reported to have low prevalence [e.g., oblivion, abnormal lying (12)], or (iv) necessary training was not available [e.g., qualitative behavior analysis (7, 12)]. Resource-based indicators that provided information on environmental conditions such as space allowance per goat and bedding material were collected.

The initial protocol was tested over multiple visits to a local farm in Iowa over a 2 week period. Two observers tested the protocol in the milking parlor and home pen to ensure the definitions accurately reflected the observations made, and the length of time required to perform the assessments. Where differences in the results between observers were observed, further training was provided to improve agreement on subsequent visits.

### On-Farm Assessments

Assessments were performed by a single assessor between March and August 2020. The assessor wore the same colored clean coveralls and used disposable boot covers and gloves between farms. Observations were manually recorded using a tablet (10.2″ iPad, 8th Generation, Apple Inc., Cupertino, CA) equipped with data collection software (REDCap, Vanderbilt University, Nashville, TN). Due to equipment malfunction after seven farm visits, data was then recorded onto printed record sheets and then manually entered onto REDCap software after completion of the farm visit.

The temperature and humidity were measured 10 min after arrival to the pens using a temperature and humidity logger (WD-20250-42; Digi-Sense, Vernon Hills, IL). Temperature and humidity ranged from −7.6 to 34.7°C with an average of 21.4°C (SD: 10.2) and 20.7% rh (relative humidity) to 80.6% rh with an average of 51.5% rh (SD: 13.7), respectively.

Intra-observer reliability was completed pre- and post-observation and was assessed by scoring 50 images of goats collected prior to farm visits (with some images collected during farm visits) and then re-examined. Percentage agreement for pre- and post-observation reliability (respectively) was as follows: 98% for ear pathology (pre- and post-observation), 94 and 98% for ocular discharge, 96 and 98% for nasal discharge, 96 and 98% for skin lesion, 92 and 90% for knee callusing, 97% for hygiene (pre-observation reliability not completed due to lack of images of goats with poor hygiene), 98% and 100% for fecal soiling, 98% and 94% for udder asymmetry, 92 and 94% for overgrown claw, 100% for horn growth (pre- and post-observation), 98 and 90% for poor hair coat condition, and 90 and 94% for body condition. Inter-observer reliability was not conducted for some indicators (e.g., queuing behavior, thermal stress, kneeling, and lameness) that showed low occurrence rates or were difficult to photograph.

### Group Assessments

The number of pens (and animals) assessed was determined at each farm visit and depended on the number of lactating goats on farm (Table 3). All pens that housed <230 lactating does were observed unless the farm had more than 600 lactating does. In this case, either one pen of goats was observed or as many pens that could be evaluated in a 2 h period. After observing all pens on the farm, the assessor chose the pen(s) to be assessed based on being representative of the farm and containing mobile, and lactating goats (i.e., not the sick pen). Note that pens were selected in this way on only three farms. The group assessments took place in the goat barn after a short acclimatization period of ~5 min. Depending on the number of animals in each pen, the group-level assessments of the goats were observed for up to 2 h. Due to inconsistencies in recording of the durations of animal observations at each farm, this information will not be reported. During this period, the assessor moved slowly along the outside rail of each pen recording observations. Once outside pen observations were complete, the assessor entered the pen and began the latency to approach test; this involved moving to a predetermined location adjacent to a pen wall and while remaining motionless and without making eye contact with the goats. Once stationary the assessor started a stopwatch and the time taken (in seconds) for the first goat to contact any part of the assessor (including recording devices) was recorded. The assessor then moved slowly throughout the pen assessing body condition and lameness. All goats within the pen were made to walk, except those that did not stand or had obvious injuries and were excluded from lameness scores. The assessor avoided contact with the goats as much as possible.

**Table 3:** The number of total pens and lactating does on-farm, the number of pens assessed (and number of does within pens) and does individually assessed in the milking parlor on each farm.

**Farm**	**Total pens on farm**	**Total lactating does on farm**	**Number of**		
			**Pens assessed**	**Does within pen(s) assessed**	**Does in milking parlor assessed**
1	1	36	1	36	36
2^a^	9	6,500	1	168	510
3^d^	1	70	1	70	67
4^b^	5	179	5	172	179
5^c^	1	142	1	142	139
6	2	178	2	178	178
7^d^	1	110	1	140	110
8	5	857	5	857	158
9	1	1,000	1	1,000	243
10	3	128	3	128	128
11	2	140	2	140	140
12	2	172	2	172	172
13	2	125	2	125	125
14	3	207	3	207	207
15	3	227	3	227	227
16	1	180	1	180	180
17^b^	7	157	7	151	157
18^e^	1	266	1	266	NA
19^c^	3	34	3	78	34
20	3	158	3	158	158
21	2	700	1	322	246
22	2	118	2	118	118
23^c^	1	180	1	185	180
24	2	91	2	92	92
25^d^	2	121	2	162	121
26	12	3,960	2	440	216
27^d^	5	204	5	204	179
28	1	145	1	145	145
29	3	144	3	144	144
30	1	187	1	187	187

a *includes separate observations of the front of 257 goats and the rear of 253 goats as the front and back of the same animal could not be observed*;

b *some goats were not observed during pen assessment*;

c *non-lactating goats were housed in the pen with lactating goats*;

d *some goats were not observed in the milking parlor*;

e *individual assessments in the milking parlor were not carried out*.

### Individual Goat Observations

The number of does on each farm assessed at the individual-level depended on the number of lactating does and is presented in Table 3. When the number of lactating does was <230, all does were assessed. For farms that had more than 230 does, the assessor observed as many does as could be observed in a 2 h period.

The assessor moved slowly between each goat, making sure to observe both sides of the head and neck region at the front of the goat and the dorsal view of the legs and both sides of the rump at the back of the goat.

### Data Management and Statistical Analysis

The data was exported from REDCap software as a comma-separated values file and used with Excel (Microsoft Corporation, Redmond, WA). The data has been presented as a mean with standard error (SE) or median with interquartile range (IQR), where appropriate. The individual- and group-level data was calculated as the number of animals displaying each indicator out of the number of animals observed per farm.

The individual assessment data from one farm was excluded from analysis as the goats were not individually observed in the milking parlor due to logistical constraints. In some instances, milking parlor layout prevented observations from being recorded (e.g., rotary parlors prevented the front and back end of the goats from being observed of the same animal) and consequently some individual assessment data were not collected on three farms. Body condition scoring and lameness data were excluded from one farm as it could not be assessed as the pen was spread across multiple buildings making clear identification of goats difficult.

A principal component analysis (PCA) biplot (based on a correlation matrix) was used to explore the relationships between the farms, and their characteristics with respect to the welfare assessment variables. Missing data (4% of the dataset) was imputed using the mean value of the variable. Heat and cold stress data were excluded from the PCA due to the variation in seasons (i.e., temperature) across farms over the study period.

## Results

Welfare assessment was performed on 30 farms in the Midwestern US and the characteristics of those farms are presented in Table 4. The number of goats assessed individually and at the group-level was 4,777 goats and 6,593 goats, respectively. The number of lactating goats ranged from 34 to 6,500 goats, with a median herd size of 158 goats (IQR = 80.8; mean ± SE: 533.9 ± 243.3 goats). The individual-level welfare assessment data are presented in Table 5 and the group-level welfare assessment data are presented in Table 6. The average latency for goats to approach the assessor was 33.6 ± 12.0 s (mean ± SE), with a range of 2.0 s to 300.0 s (note that the test ended at 300 s).

**Table 4:** Characteristics of 30 dairy goat farms in the Midwestern United States.

**Farm characteristics**	**Value**
Breeds (No. of farms)^*^	
Saanen	28
Alpine	28
American LaMancha	18
Anglo-Nubian	11
Toggenburg	10
Oberhasli	6
Sable	2
Kiko	1
Feed space/goat (mean ± SE; min-max; ft)	1.0 ± 0.1 (0.3–1.8)
Total space allowance/goat (mean ± SE; min-max; ft^2^/goat)	108.3 ± 43.7 (14.0–1282.0)
Indoor (mean ± SE; min-max; ft^2^/goat)	29.3 ± 4.8 (7.4–132.7)
Outdoor (mean ± SE; min-max; ft^2^/goat)	78.8 ± 41.2 (0–1178.7)
Type of feed (No. of farms)^*^	
Hay	27
Grain/concentrate	27
Fermented forage (e.g., silage)	7
Total mixed ration	3
Fresh cut grass	2
Corn	1
Bedding material	
Straw (No. of farms, %)	24 (80.0)
Corn husks (No. of farms, %)	3 (10.0)
Soy fodder (No. of farms, %)	1 (3.3)
Straw, wood shavings, corn husks (No. of farms, %)	2 (6.7)
Milking procedure	
Mechanical (No. of farms, %)	28 (93.3)
Hand-milking (No. of farms, %)	2 (6.6)
Access to outdoor space (No. of farms, %)	22 (73.3)
Outdoor space surface	
Earthen (No. of farms, %)	19 (86.4)
Pasture (No. of farms, %)	13 (59.1)
Concrete (No. of farms, %)	6 (27.3)
Rock (No. of farms, %)	2 (9.1)
No. of permanent staff (mean ± SE; min-max)	6.3 ± 0.9 (1.0–25.0)

* *farms provided more than one type of feed and raised more than one breed of goat*.

**Table 5:** *Individual-level* welfare indicators observed for 4,524 goats on 30 farms across the Midwestern United States during on-farm welfare assessment at milking.

**Indicator**	**Number of**		**Variation in indicator occurrence (% of goats) across farms**		
	**Goats (%)**	**Farms (%)**	**Median**	**IQR**	**Maximum**
Any ear pathology	361 (8.0)	23 (85.2)^Ψ^	6.5	1.5–12.7	38.6
Frostbitten ears	151 (3.3)	17 (65.4)^Ψ^	1.1	0–3.1	29.3
Torn ears	94 (2.1)	17 (65.4)^Ψ^	1.1	0–3.6	6.7
Missing ear tags	110 (2.4)	18 (69.2)^Ψ^	0.8	0–3.4	11.7
Infected ear tags	6 (0.1)	5 (19.2)^Ψ^	0.5	0–2.5	3.9
Ocular discharge	132 (2.9)	22 (81.5)*	1.6	0.8–3.8	17.6
Nasal discharge	313 (6.9)	25 (92.6)*	3.5	1.4–8.5	38.5
Skin lesions^η^	427 (8.9)	26 (96.3)*	10.4	4.5–14.5	40.0
Any knee callusing^Ω^	3,657 (80.9)	26 (100)^Ψ^	96.8	82.8–99.1	96.8
Mild^Ω^	2,516 (55.7)	26 (100)^Ψ^	63.8	53.4–75.4	63.8
Severe^Ω^	1,141 (25.2)	26 (100)^Ψ^	29.2	14.9–41.6	53.1
Poor hygiene (dirty)^Ω^	674 (14.9)	24 (82.8)^¥^	9.6	3.3–23.7	43.1
Fecal soiling^Ω^	157 (3.5)	24 (82.8)^¥^	1.6	0.5–6.3	20.9
Udder asymmetry^Ω^	147 (3.3)	24 (82.8)^¥^	2.9	1.2–4.8	11.1
Any claw overgrowth^Ω^	2,325 (51.4)	28 (96.6)^¥^	48.6	20.6–75.6	98.3
Mild^Ω^	1,527 (33.8)	28 (96.6)^¥^	30.0	14.8–46.6	67.8
Severe^Ω^	798 (17.7)	21 (72.4)^¥^	6.6	0–28.7	69.9

*Total number of goats observed for indicators: ^Ω^ = 4,520, ^η^ = 4,777*.

Data was excluded from:1 farm^¥^, 3 farms*, 4 farms^Ψ^.

**Table 6:** *Group-level* welfare indicators observed for 6,593 goats on 30 farms across the Midwestern United States during on-farm welfare assessment.

**Indicator**	**Number of**		**Variation in indicator occurrence (% of goats) across farms**		
	**Goats (%)**	**Farms (%)**	**Median**	**IQR**	**Maximum**
Queuing at feed rack(s)^Ω^	247 (6.8)	22 (75.9)*	5.0	0.4–11.7	35.6
Queuing at drinking place(s)^Ω^	73 (2.0)	14 (50.0)^¥^	0.0	0.0–2.9	11.1
Horn growth					
Horns	79 (1.2)	11 (36.7)	0	0–0.9	16.0
Scurs	365 (5.5)	28 (93.3)	2.9	1.8–8.0	24.2
Poor hair coat condition	545 (8.3)	30 (100)	6.9	4.9–12.1	27.3
Thermal stress					
Cold stress	4 (0.1)	2 (6.7)	0	0	4.3
Heat stress	243 (3.7)	12 (40)	0	0–3.6	50.8
Kneeling					
In pen	17 (0.3)	8 (26.7)	0	0–0.2	2.3
At feed rack(s)	43 (0.7)	11 (36.7)	0	0–0.6	5.7
Body condition					
Overweight	256 (3.9)	26 (92.9)^¥^	4.1	1.7–5.7	15.2
Underweight	264 (4.0)	26 (92.9)^¥^	2.8	1.0–9.2	22.9
Lameness	99 (1.2)	22 (75.9)*	1.2	0.5–2.3	4.3

*Total number of goats observed for indicators: ^Ω^ = 3,606*.

Data was excluded from: 1 farm*, 2 farms^¥^.

**Figure 1:**
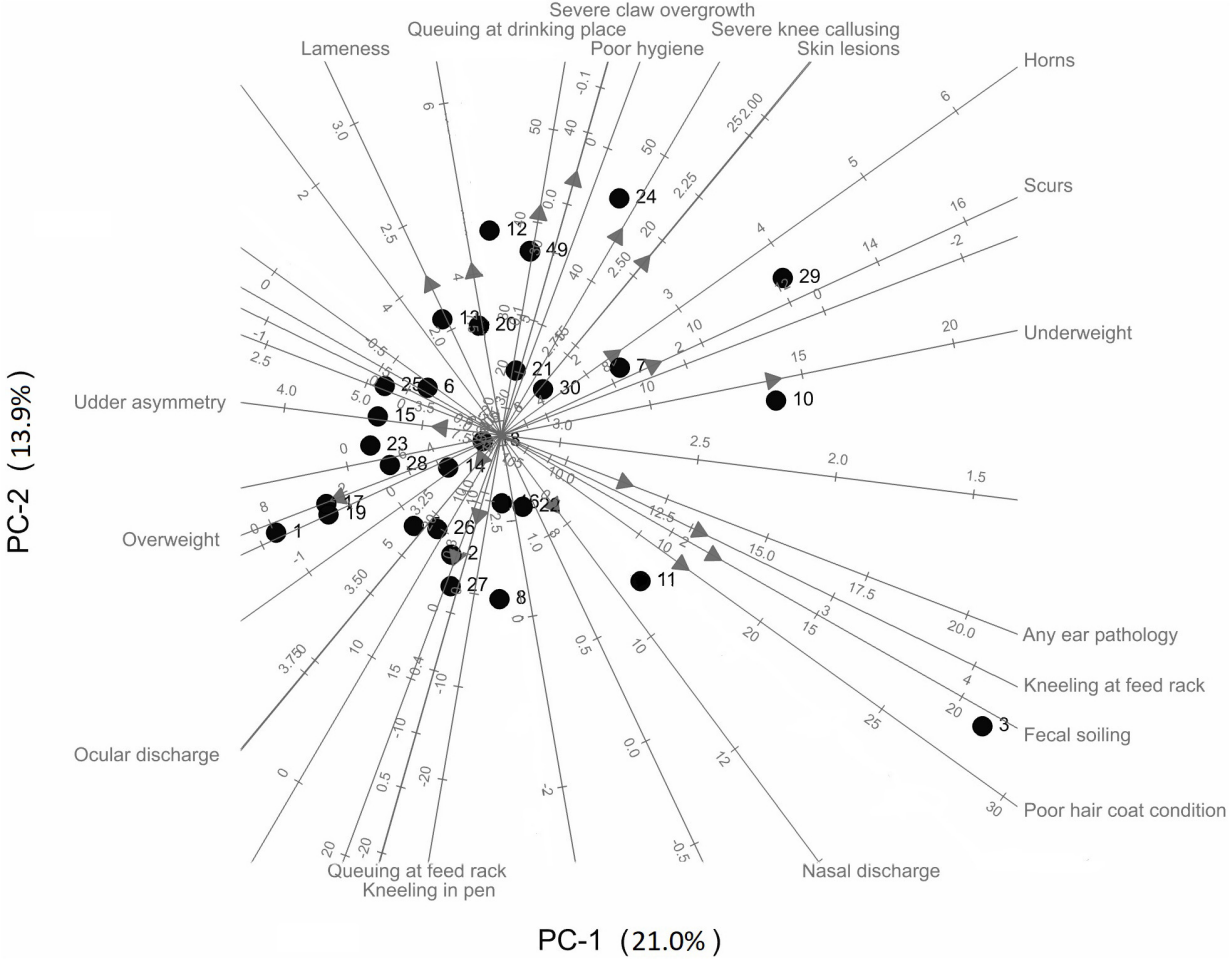
Principal components biplot of welfare indicators of dairy goats across 30 farms in the Midwestern United States.

Results of a PCA biplot on the welfare assessment data from each farm are shown in Figure 1. The overall welfare state of the goats on each farm was described using 19 animal-based indicators (latency to approach test, and heat and cold stress were not included). The first 2 dimensions of the PCA (PC-1 and PC-2) explain 34.8% of the variation. For each variable, the direction of its biplot axis is indicated by an arrow. Axes of welfare indicators that are close to one another (and in the same direction) indicate these variables are positively correlated (e.g., severe claw overgrowth and poor hygiene); axes with arrows in opposing directions indicate negative correlations (e.g., overweight and horns), and perpendicular axes indicate no correlation (e.g., ocular discharge and any ear pathology). The individual farms are represented by points. The predicted value of a welfare indicator for a farm is given by projecting the point onto the axis (i.e., drawing a perpendicular line from the point to the axis). Thus, farms that cluster together (e.g., Farms 17 and 19) are predicted to have similar characteristics with respect to the welfare indicators, and those far apart (Farms 17 and 29) are predicted to be dissimilar.

Farms with a high number of goats that have horn growths (scurs or horns), ear pathologies, fecal soiling, poor coat condition, are underweight, and kneel at feed racks are on the right side of Figure 1 (e.g., Farms 3, 7, 10, 11, 24, and 29). Conversely, farms with a low number of goats with these welfare issues are scattered on the left side of Figure 1 (e.g., Farms 1, 17, and 19). Farms scattered near the top of Figure 1 have a high number of goats that are lame, have severe claw overgrowth, perform queuing at the drinking place, experience heat stress, poor hygiene, severe knee callusing and skin lesions, but a low number of goats with that experience cold stress, have nasal discharge, perform kneeling in the pen and queuing at the feed rack (e.g., Farms 4, 9, 12, and 24).

## Discussion

The objective of this study was to perform welfare assessment of dairy goats on 30 farms across the Midwestern US and identify the most prevalent welfare issues. Based on the results of our study, the most prevalent welfare issues observed were knee callusing, claw overgrowth, poor hygiene, skin lesions, poor hair coat condition, and ear pathologies. The collected data was processed and then provided to the producers in the form of benchmarking reports. These reports contained the range of values across farms, the median value, and each farms' average for the welfare indicators. Thus, producers were able to visualize their farms' comparative success (or failure) to the other farms in the study. It was hypothesized that provision of benchmarking reports would encourage producers to alter their farm practices to improve goat welfare in the areas identified as being deficient in comparison to the other farms. Farm visits to conduct secondary welfare assessment and evaluate the effect of the benchmarking reports was delayed due to COVID-19 restrictions on travel.

On-farm welfare assessment of dairy goats has been previously conducted in Europe (7, 12, 14), the United Kingdom (6), and more recently, Mexico (23); however, to the authors' knowledge, these are the first data on dairy goat welfare assessment on farms across the Midwestern US. In 2017, Europe produced 15% of global dairy goat milk production, compared with 4% from the Americas (24). There are differences (and similarities) that exist between North American and European dairy goat industries and associated farming practices (e.g., intensive vs. semi-intensive farming, breeds raised, pain management for painful husbandry practices). In Europe, dairy goat production is highly specialized for milk production likely associated with the higher demand for goat milk products; whereas dairy goat production is comparatively less well-developed, and relatively small by global standards in the US (24). Information on dairy goats in the US is limited due to the viewpoint that goats are a minor species in comparison with cattle, creating issues for farmers, veterinary practitioners, and policy makers (24). Although there are large-scale, commercial dairy goat farms in operation (e.g., 9,000-goat herds), the majority are still small (25). Recent data from the National Animal Health Monitoring Survey (NAHMS), Goat Study 2019 shows that the average herd size across the US is approximately 20 goats (26). For a review of recommendations on dairy goat kid husbandry practices under intensive production systems in Canada, US and France please refer to Bélanger-Naud and Vasseur (64).

Mild or moderate knee calluses are a common occurrence among dairy goats [99.3% of 575 goats (7)], and can reflect the type(s) of surface or amount of bedding available, but it is the severity of knee callusing (i.e., thickness, full width of the knee, broken skin) that may be a welfare concern. Severe knee calluses can be indicative of excessive kneeling, insufficient or inadequate bedding (discussed later) and may be associated with lameness (6). However, the PCA in the present study, showed a negative correlation between severe knee callusing and kneeling in the pen (and only a weak positive correlation with lameness). Additionally, kneeling at the feed rack appeared to show no relationship with severe knee calluses (or kneeling in the pen). This result contradicts our assumption that increased time spent on the knees would result in knee calluses. Anzuino et al. (6) reported that 79.2% of 24 farms in the UK had goats kneeling at the feed trough, but that this was not correlated with lameness. Although observing kneeling behavior on farms is a valid and feasible indicator of discomfort at the feed trough, whether it has good intra- and inter-reliability remains unknown (10). In the present study, the assessor observed the goats in the home pen for up to 2 h, which may not have been enough time to adequately sample kneeling behavior. Further, the assessor observed the goats during two different time periods (i.e., before or after milking), which may affect our ability to directly compare differences, but was utilized for feasibility in relation to assessing multiple farms per day. We observed mild knee calluses in just over half of the animals assessed with a further 17.7% of goats with severe knee calluses. Severe knee calluses have been reported previously and range from 8.9 to 18.3% (6, 12). The relatively high proportion of goats with severe knee calluses in the present study may be associated with bedding-related factors such as type, depth, dirtiness, or wetness of the bedding. The majority of the farms in this study used straw bedding, similar to those involved in the study of Anzuino et al. (6), which demonstrated that severe knee calluses were positively correlated with dirty limbs. Bedding that is wet, dirty or with poor drainage can increase the risk of developing skin lesions in swine (27) and dairy cattle (28, 29). Cows bedded on sand presented lesions of lower severity and were less dirty than those bedded on straw (30). Future research on the effect of bedding or lying surfaces on hock or knee calluses or skin lesions for goats is required to improve bedding management and goat welfare.

Severely overgrown claws typically result from a lack of wear of the claw or insufficient foot trimming. To reduce the risk of welfare problems such as lameness, which correlates with claw overgrowth (6, 31, 32), trimming should be undertaken at least twice yearly in intensive farms, where movement is limited (10). In the present study, we observed relatively low rates of severe claw overgrowth (17.7%), compared with previous studies, which ranges from 16.8 to 55.5% (6, 7, 12, 14, 32). Anecdotally, producers may be hesitant to perform frequent claw trimming as they believe that this encourages growth. More research is required demonstrating the benefits of regular foot trimming practices in preventing welfare issues such as lameness (discussed below). In addition, the provision of abrasive surfaces in the home pen or parlor that may encourage natural hoof wear should be considered. Further, environmental enrichment (e.g., rocks) can improve welfare outcomes by allowing for expression of natural behavior although not validated.

Hygiene or cleanliness is considered a valid indicator of welfare in dairy cows (29, 33), poultry (34) and goats (6, 12). Goats generally prefer not to lie in wet bedding, and goat feces is dryer than cattle; therefore, goats with poor hygiene may be indicative of poor environmental cleanliness and management practices (e.g., inadequate bedding management) (10). At high ambient temperatures (e.g., 38.0–39.5°C), goats generally show increased water intake and experience diuresis (35), which may result in a wetter environment; therefore, a higher ambient temperature may explain the increased rates of dirtiness with heat stressed goats. Increased lying duration has been reported in goats experiencing high ambient temperatures and with restricted water supply (36). A further explanation for a relationship between heat stress and poor hygiene is that to reduce the negative effects of heat stress, goats may lie in wet bedding to increase heat loss. Cows spend less time lying down during periods of heat stress to expose more body surface area for evaporative cooling (37); however, cows will actively avoid wet bedding to reduce the effects of conductive heat loss when experiencing cold temperatures (38). Observations of poor goat hygiene range from 2.4 to 36.4% (6, 7, 12, 14). In the present study, we observed 14.9% of goats had poor hygiene. It is important to note that the definition used in the present study included the presence of any fecal material (or dirt) and therefore the number of animals in the study with poor hygiene may be over-represented. The wide variation in the amount of goats with poor hygiene observed across studies may be associated with how the body areas were classified; for example, whether separate anatomical areas were hygiene scored (6, 12) or collective regions were scored together (i.e., rump, thighs, udder, and rear legs) as has been done in the present study.

Poor hair coat condition has been demonstrated as a reliable and valid indicator of welfare in goats; goats with poor hair coat condition had lower body condition (underweight), mineral deficiencies, presence of ectoparasites, and higher prevalence of abnormal lung sounds (11). Poor hair coat condition can be defined as uneven or shaggy and matted, that is frequently longer than normal, whereas a normal coat is shiny, smooth and adheres to the body's surface (11). We observed 8.3% of goats with poor hair coat condition, which is far lower than the reported ranges in Europe of 22.9 to 24.1% (12, 14). The comparatively lower rate of poor hair coat condition is likely associated with differences in sampling methodology. Battini et al. (12) and Can et al. (14) selected the pens with the worse welfare conditions (e.g., high stocking density, horned and hornless animals together, limited access to resources), which likely captured a greater number of animals with poor hair coat condition, compared with the present study, which used a different strategy.

Ear pathologies were observed on farms in the present study. The most common ear pathologies were characterized as damage associated with ear tags (either missing or torn ears), and frostbite. The majority of the farms involved in this study used ear tags as a form of identification (18/30; Hempstead et al., unpublished data). Incorrect placement of ear tags that are not in the center of the ear may result in inflammation or ear tears (6, 39). Ear tags may be ripped out as goats move their heads in and out of the feed troughs. In the present study, 2.1% of 4,524 (94 goats) goats had ear tears, which is in line with Anzuino et al. (6), who reported that 6.2% of 1,520 (~94 goats) goats had ear tears. Frostbitten ears are generally the result of extended exposure to low temperatures when the animals are first born. Care must be taken to ensure newborns are dried (especially the ears and feet) shortly after birth, and/or by moving newborn kids to temperature controlled environments to reduce the incidence of frostbitten ears (40). The extent of pain or discomfort associated with ear tears and frostbite is not well-understood and requires further investigation.

Skin lesions such as abscesses, swellings, or broken skin and hair loss can be indicative of many health issues including caseous lymphadenitis (CL), or other dermal skin infections, ectoparasites and tissue injury from animals with horns, or environmental structures (40–42). There is a wide range of prevalence rates of skin lesions from 0.3 to 35.5% (6, 7, 12, 14), and our data appears to be on the lower end of the range (8.9%); this may have multiple explanations. First, there were differences in research methodologies between studies: skin lesions were categorized into anatomical regions of the body in earlier studies, whereas we evaluated skin lesions together without specifying the location on the body. Sampling strategies across studies also differed as we observed the goats in the parlor at the speed they were milked, whereas Can et al. (14) and Battini et al. (12) observed the goats restrained whilst in the pens. The best location for assessing skin lesions on dairy goats requires further validation. Second, there are likely differences in management practices such as utilization of a vaccination program for CL, minimization of pen structures that can cause skin lesions (e.g., protruding wire or sharp objects), treatment for ectoparasites or disbudding practice (discussed later).

Body condition scoring evaluates the level of muscle and fat development and is a reliable and valid method of monitoring fluctuations in fat reserves (10, 43, 44). A numerical rating scale of 5 points is commonly used across ruminant species (7, 45, 46). Until recently, the most accurate form of body condition scoring goats involved palpation of the lumbar and sternum regions due to differences in the amount of visceral and subcutaneous fat deposits with other species (47); however, valid and reliable BCS can be conducted from observations of the rear of the animal either in person or from digital photos (43, 44), which removes the need for individual restraint. Furthermore, identification of animals experiencing extreme nutritional deficiencies (e.g., overweight/too fat or underweight/too thin), compared with assigning a score (i.e., from 1 to 5), may reduce the time required and hence improve on farm feasibility and reliability (10). Underweight animals may have decreased feed intake where their energy expenditure exceeds nutritional status, which may reflect an inadequate feed supply or increased energy output, whereas overweight animals are generally the result of overfeeding or excessive confinement (5). In the present study, the amount of underweight and overweight goats appeared similar (4.0 and 3.9%, respectively), indicating that feed management is an area of potential improvement for farm managers. However, some caution should be taken when interpreting our results as due to the sampling strategy (i.e., sampling animals in the home pen where animals are free to move around), some animals may have been missed or counted twice. Other studies have reported overweight goats ranging from 2.7 to 18.2% and underweight goats ranging from 3.4 to 13% (6, 12, 14). The PCA shows that there was a positive correlation between underweight, fecal soiling, and poor hair coat condition, which may be associated with disease. Paratuberculosis or Johne's disease is a chronic wasting disease that affects ruminants and causes persistent diarrhea, progressive weight loss and may lead to death (48, 49).

Disbudding is a common husbandry procedure carried out to prevent horn growth that can result in injuries [see review by (50)]. If incomplete disbudding is performed (i.e., not enough horn bud tissue removed), then scurs will likely result. Scurs are partial horn regrowth's that are not fused to the frontal bone of the skull. Animals that have been disbudded unsuccessfully and have scur development or not disbudded at all and have horns, can have injurious interactions with conspecifics (51). Furthermore, horned and hornless goats show differences in their behavior toward each other, in that horned goats display more threat behavior compared with hornless goats, which attack others more frequently (52, 53). Previously reported rates of scurs range from 6.4 to 12.7% (6, 12, 14) and a single study reported 1.5% of goats assessed (~23 of 1,520 goats) were not disbudded and had horns (6). We observed scurs and horns at a rate of 5.5 and 1.2%, respectively, which showed a positive correlation in the PCA. Together, these results demonstrate firstly, the difficulty in preventing horn regrowth in goats, and secondly, deficiencies in adequate training and practice of the operators performing disbudding, which is an area gaining attention for dairy calves (54, 55), but is still required for the dairy goat industry. In addition, extended iron application can cause brain injury in goat kids (56), which may mean that disbudding operators use less application time than required to adequately destroy the horn buds to avoid brain damage. Therefore, alternatives to cautery disbudding that reduce or eliminate pain and brain injury should be investigated.

Lameness is a debilitating condition that is associated with pain (57) and is a common issue on dairy goat farms with a range of 9.1 to 24% (6, 31, 32) and 1.7 to 3.1% in the UK and Europe, respectively (7, 12, 14). Lameness can be caused by multiple factors including overgrowth of claws (with or without conformational changes of the hoof) associated with infrequent hoof trimming or lack of natural wear, or diseases such as interdigital dermatitis, foot rot, foot lesions or caprine arthritis encephalitis (31, 32, 58). Furthermore, lameness is a useful behavioral indicator of pain in sheep (59, 60) and cattle (57, 61, 62), but studies on pain associated with lameness in goats are limited. Scoring systems for evaluating lameness in goats typically use a 4-point scale (7, 31, 63). Although, more recently, Deeming et al. (65) developed a 5-point scoring system to identify initial signs of lameness in goats (i.e., uneven gait) allowing for early intervention. Gait scoring individual animals was impractical in the present study due to the high number of animals observed, therefore only goats that were obviously lame were quantified. We observed a relatively low number of lame goats (1.2%), compared to the other studies described. Apparent differences in lameness rates across studies may be associated with different management practices, such as frequency of hoof trimming, the availability of hard surfaces or outdoor spaces to encourage natural wear of claws and how lameness was evaluated (10). Anzuino et al. (6) assessed lameness whilst the goats were exiting the milking parlor, whereas the other studies, including the present study, assessed lameness in the pens, where the soft bedding material may have concealed those goats with minor or moderate lameness (6). Additionally, the use of level surfaces (i.e., flat) for gait scoring may provide the most accurate reflection of lameness (57), which may not always be present. Another factor affecting the rates of lameness observed in the present study is that due to the sampling strategy (as for BCS), some animals may have been missed or counted repeatedly due to sampling in the home pen with animals able to freely move around.

We acknowledge that our study was not without limitations. To our knowledge there were no publicly available databases of dairy goat farms within the Midwestern US that we could access, thereby preventing random selection of farms. Therefore, farms included in this study were self-selected meaning that the data collected may not be representative of the wider dairy goat population in the Midwest US as a whole. However, our study was able to provide useful education resources and information on goat well-being for those producers that were involved. In follow-up visits, we can evaluate whether the benchmarking reports affected dairy goat well-being. We acknowledge that there was likely an effect of how the data was collected in separate sampling periods on our results; for example, queuing behavior was observed prior to milking on some farms and following milking on others and motivation to access the feed rack was likely affected. Further, the time of feed distribution relative to assessment of the home pen was not recorded, which may have also influenced the level of queuing behavior observed as fresh feed was likely not fed out at the same time across farms. Ideally, all assessments would have been completed at the same time of the day across farms, but this was not possible in the present study due to logistical restraints of time and personnel. The amount of time that the goats were observed in the pen was not recorded consistently, however, these times generally differed between farms, due to the difference in the number of animals on each farm. This likely affected the number of animals across farms observed for the various behavioral indicators assessed (e.g., queuing, kneeling). In addition, the difference in time spent in the milking parlor observing individual goats likely impacted on our results, as goats that were slower to enter the milking parlor for some reason (e.g., less dominant, sick, or injured), may have been missed. There is need for a more standardized protocol in relation to observations around feeding times and morning or afternoon milking sessions as outlined above. Future studies on welfare assessment are required that utilize a greater sample of goat farms (than the present study) and those that are randomly selected, to achieve a more accurate reflection of areas of dairy goat welfare deficiency in the Midwestern US.

In conclusion, our developed protocol for evaluating dairy goat welfare on farm in the Midwestern US identified areas of deficiency including knee calluses, claw overgrowth, poor hygiene, skin lesions, poor hair coat condition and ear pathologies. Further, using this protocol to assess a combination of welfare indicators, we have identified farms that may require changes to husbandry practices or the environment in order to improve goat welfare. The results of this research can be used by producers to improve dairy goat welfare and by researchers to continue evaluating welfare assessment on-farm in the Midwestern US.

## Data Availability Statement

The original contributions presented in the study are included in the article/supplementary material, further inquiries can be directed to the corresponding author/s.

## Ethics Statement

The animal study was reviewed and approved by Institutional Animal Care and Use Committee at Iowa State University (Protocol number: IACUC-18-341). Written informed consent was obtained from the owners for the participation of their animals in this study.

## Author Contributions

MH, TL, JS, LS, and PP: conceptualization and methodology. MH and VC: formal analysis. MH and TL: investigation. MH: writing—original draft preparation. MH, TL, JS, LS, VC, and PP: writing—review and editing. PP and JS: funding acquisition. All authors contributed to the article and approved the submitted version.

## Conflict of Interest

The authors declare that the research was conducted in the absence of any commercial or financial relationships that could be construed as a potential conflict of interest.
